# A Dangerous and Unrecognized Interaction of Apixaban

**DOI:** 10.7759/cureus.19688

**Published:** 2021-11-18

**Authors:** Hussam Ammar, Rukma R Govindu

**Affiliations:** 1 Internal Medicine, MedStar Washington Hospital Center, Washington, USA; 2 Internal Medicine, The University of Texas Health Science Center at Houston, Houston, USA; 3 Internal Medicine, The University of Texas Mcgovern Medical School at Houston, Houston, USA

**Keywords:** hiv, cobicistat, cyp3a4 inhibitor, cytochrome 450, drug interaction, apixaban, vascular procedure

## Abstract

Direct oral anticoagulants (DOACs) drug-to-drug interactions are underrecognized by clinicians. Apixaban has cytochrome 450 (CYP) mediated metabolism (primarily by CYP3A4). Strong inducers and inhibitors of this enzyme may cause variations in the blood level of apixaban. This report presents a patient who received a femoral artery stent and developed a large retroperitoneal hemorrhage after she was prescribed apixaban in addition to her antiretroviral therapy (AVT) regimen that included cobicistat, a strong CYP3A4 inhibitor. The patient was managed conservatively, and a repeat computed tomography scan in a subsequent admission revealed near resolution of the hematoma. The treating physicians realized that apixaban should not be prescribed with a potent CYP3A4 inhibitor like cobicistat and discontinued it.

## Introduction

Direct oral anticoagulants (DOACs) have emerged as safe and effective alternatives to warfarin. They are more convenient for the patients and physicians as they do not require frequent blood monitoring and are given safely in fixed doses. They also have fewer drug-food and drug-drug interactions. A systematic review of the published literature of adverse events associated with drug-drug interactions with DOACs throughout 2020 documented only 23 reports. The majority were case reports (n = 20) documenting a single adverse event resulting from a suspected DOAC drug-drug interaction, while the remaining papers were a case series (n = 1) and cohort studies (n = 2). The most described adverse drug reactions were adverse drug reactions related to hemorrhage or thrombosis [1].

## Case presentation

**Figure 1:**
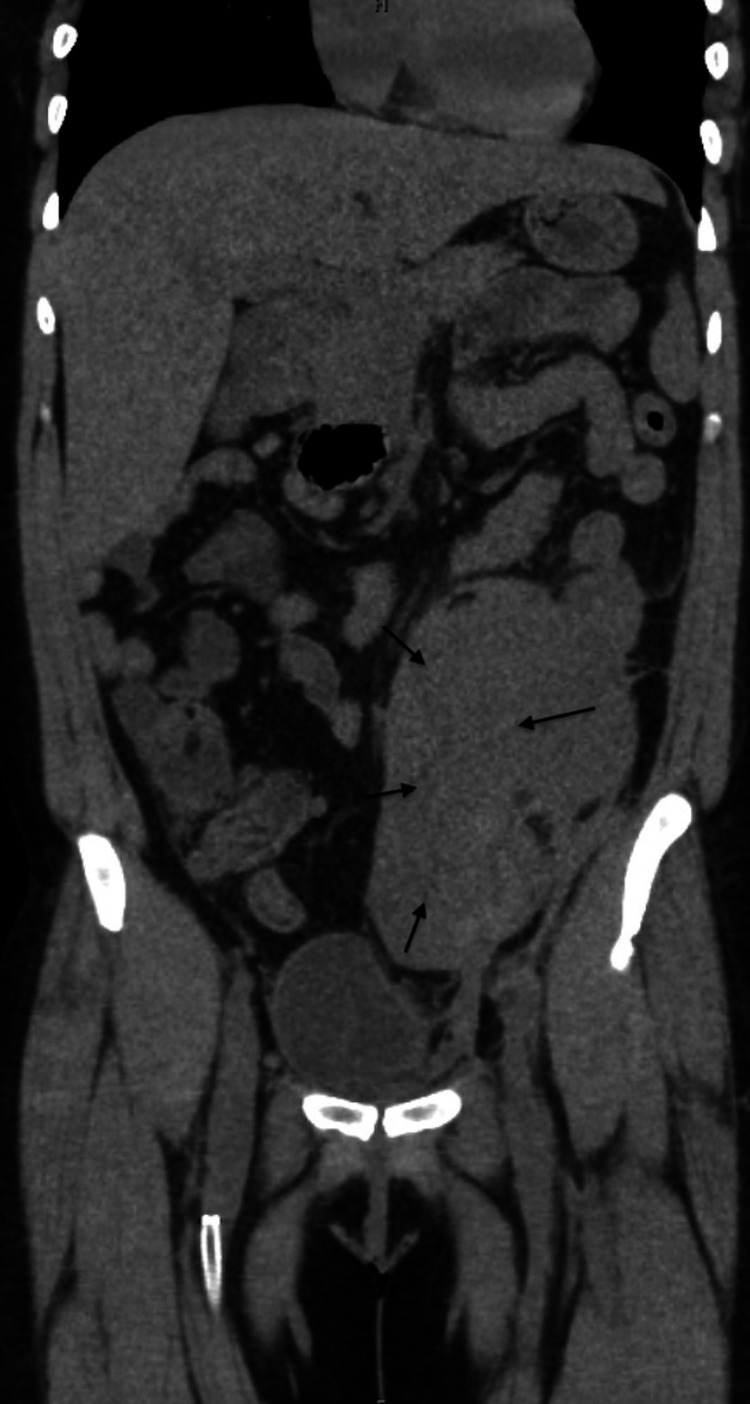
CT coronal image Retroperitoneal hematoma "arrows"

**Figure 2:**
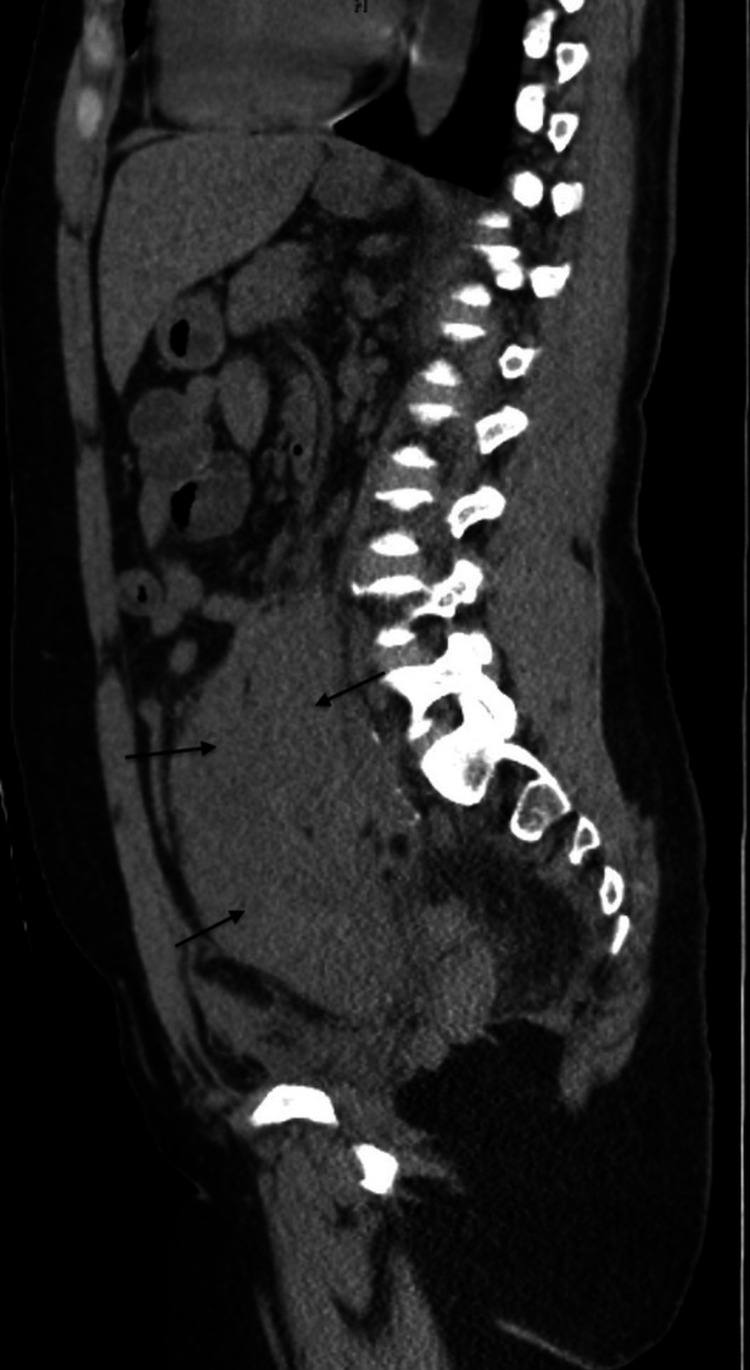
CT sagital image Retroperitoneal hematoma " arrows"

A 40-year-old woman was admitted for evaluation of critical limb ischemia of the right leg. Medical history is significant for human immune deficiency virus (HIV) infection with undetected viral load, coronary artery disease, peripheral vascular disease, and polysubstance abuse. Vascular surgery performed bilateral lower extremity angiogram, femoral artery angioplasty, and a femoral artery stent. The postoperative course was uneventful. Apixaban 2.5 mg twice daily was added to her medication regimen of clopidogrel, Darunavir/cobicistat (Prezcobix), abacavir/dolutegravir/ lamivudine (Triumeq), atorvastatin, isosorbide dinitrate, and hydralazine. The patient returned one-week post-discharge with low back pain and a computed tomography (CT) scan demonstrated a large retroperitoneal hematoma (Figures 1-2).

Her hemoglobin level on admission was 9.2 g/dl, down from a discharge level of 11.9 g/dl. Her serum creatinine was stable at 0.8 mg/dl. On arrival to ED, blood pressure was 130/69 mm of Hg, the heart rate was 87 beats per minute, respiratory rate was 18 breaths per minute, and the temperature was 37.4 °C. The patient was managed conservatively, and both her hemoglobin and vital signs remained stable throughout her admission. She was discharged on apixaban and clopidogrel as recommended by the vascular team. The patient was readmitted four weeks later with urosepsis. A follow-up CT abdomen revealed a resolving retroperitoneal hematoma. The patient reported that during her outpatient management she had only been compliant with her antiretroviral therapy (ART) and not with her antiplatelet or anticoagulant medication (clopidogrel and apixaban). Her anticoagulant regimen was changed to enoxaparin out of concern with potential drug-drug interaction with apixaban and cobicistat, and the team continued clopidogrel. This warning was identified by the electronic medical records.

## Discussion

Retroperitoneal hemorrhage is a rare complication of procedures that require femoral access, such as cardiac catheterization and peripheral vascular procedures. Anticoagulation is one of the factors that increase the risk of this complication [2]. Rivaroxaban and apixaban have cytochrome 450 (CYP) mediated metabolism (primarily by CYP3A4). Also, all DOACs, including apixaban, are substrates of the efflux transporter P-glycoprotein (P-gp) which regulates the elimination of DOAC from the gastrointestinal lumen. Potent CYP3A4 and P-gp inducers may reduce the plasma concentration of apixaban and cause thrombosis while potent inhibitors may increase the plasma concentration of apixaban and cause bleeding [1,3]. Cobicistat is a potent CYP3A inhibitor and P-gp inhibitor. It is used as a pharmacokinetic enhancer to increase the plasma level of ART. In a study of healthy volunteers, it was found to significantly increase the plasma level of apixaban, edoxaban, and rivaroxaban generating area under curve time ratio (AUCR) of 1.67-2. This level of AUCR requires a dose reduction of 30-50% of the dose of these three DOACs when are given with cobicistat-containing drugs [4].

A literature review and an analysis of the World Health Organization database of spontaneous safety reports of drug interaction with apixaban registered 10 case reports [3]. Five cases were pharmacokinetic interactions through inhibitors or inducers of CYP3A4 and or P-gp with a wide range of bleeding and thrombosis side effects. Three cases were pharmacodynamic interactions through additive pharmacologic effects. Two cases were a combination of both. Two case reports, caused by a pharmacodynamic interaction, described cardiac tamponade after apixaban and ibrutinib (platelet dysfunction) co-administration. The last pharmacodynamic interaction involved a selective serotonin reuptake inhibitor (SSRI) alone. For the combined pharmacokinetic and pharmacodynamic interactions, one case involved an SSRI that induced platelet dysfunction and CYP3A4 inhibition, and another case involved both an SSRI (platelet dysfunction) and a CYP3A4/P-gp inhibitor [3].

A systematic review of reports of DOACs' drug-to-drug interactions revealed only 23 publications after a decade of use. The most frequently reported bleeding events in these case reports involved the combinations of amiodarone and dabigatran (via inhibition of P-gp) and ritonavir and rivaroxaban (via inhibition of both P-gp and CYP3A4). The most frequently reported cases for thrombosis involved the antiepileptic drugs phenobarbital, phenytoin, and carbamazepine (via induction of P-gp and/or CYP3A4) [1]. A case series of six patients reported successful use of a reduced dose of apixaban at 2.5 mg twice daily for HIV patients on ritonavir or cobicistat-boosted ART for indications of deep venous thrombosis and atrial fibrillation with no bleeding complications or recurrence of thrombosis [5]. Our case is different from this case series as apixaban was prescribed in the setting of femoral artery intervention, which has a higher bleeding risk than patients with deep venous thrombosis or atrial fibrillation. 

Various countries have different recommendations on prescribing apixaban with potent CYP3A4 inhibitors. The Food and Drug Administration (FDA) approved labeling in the United States for apixaban which recommends the dose to be decreased to 2.5 mg twice daily when it is co-administered with drugs that are strong dual inhibitors of CYP3A4 and P-gp. The labeling stated that in patients already taking apixaban at a dose of 2.5 mg daily, avoid coadministration with strong dual inhibitors of CYP3A4 and P-gp [6]. Health Canada recommends against combining apixaban and strong dual CYP3A4 and P-gp inhibitors. The European regulators do not recommend prescribing P-gp/strong CYP3A4 inhibitors with apixaban [7].

## Conclusions

Clinicians should be more aware that DOACs, like apixaban, are not without serious drug-to-drug interactions that can have life-threatening consequences. It is prudent to avoid prescribing a combination of apixaban (albeit at a lower dose) with dual strong CYP3A4 and P-glycoprotein inhibitors like cobicistat, particularly in the setting of a recent vascular procedure.
